# Correspondence

**Published:** 1874-08

**Authors:** 


### Correspondence.

———————, August 15, 1874.

Dear Sir :—

Since the twelfth of May, 1874, all well informed editors of this metropolis, and generally all over the State, have known that we have a new medical law.

On the 9th of June it was the subject of a formal resolution by the Erie County Medical Society at Buffalo. On the 25th of June, was publicly discussed by the New York Medico-Legal Society, and was published in full, with comments, by the New York Medical Journal July 1st, 1874 ; but a semi-monthly Medical Journal, under date of 15th of July, in a very obscure paragraph, thus treats it :

"Just before the adjournment of the State Legislature the following law was passed, but we have not learned whether it has or has not received the signature of the Governor." Then follows a copy of the law. What name will duly express such confessed ignorance in a metropolitan medical editor? Is he an editorial Rip Van Winkle? Or an Aminadab Sleek? not affording to attend to the wants of his neighbors, because he was bound to furnish fine-tooth combs and flannel shirts to the little Africans. We incline to the latter, because on the same page he gives us a long story about Chinese physiology, and a romantic newspaper case of electrolysis.

An Editorial Aminadab Sleek !

